# Supplementary material for: Conditional chemoconnectomics (cCCTomics) as a strategy for efficient and conditional targeting of chemical transmission
Source: eLife. 2024 Apr 30;12:RP91927. doi: 10.7554/eLife.91927 (PMC11060718; doi:10.7554/eLife.91927)
Supplement: Figure 3—source data 3. [file elife-91927-fig3-data3.zip › Figure 3 source data3 part 1.zip!/Figure 3-Source data 3-part1/1082-9-CRISPResso_Report_zod2ewbs.zip!/CRISPResso_on_1082_9/1a.Read_barplot.pdf]

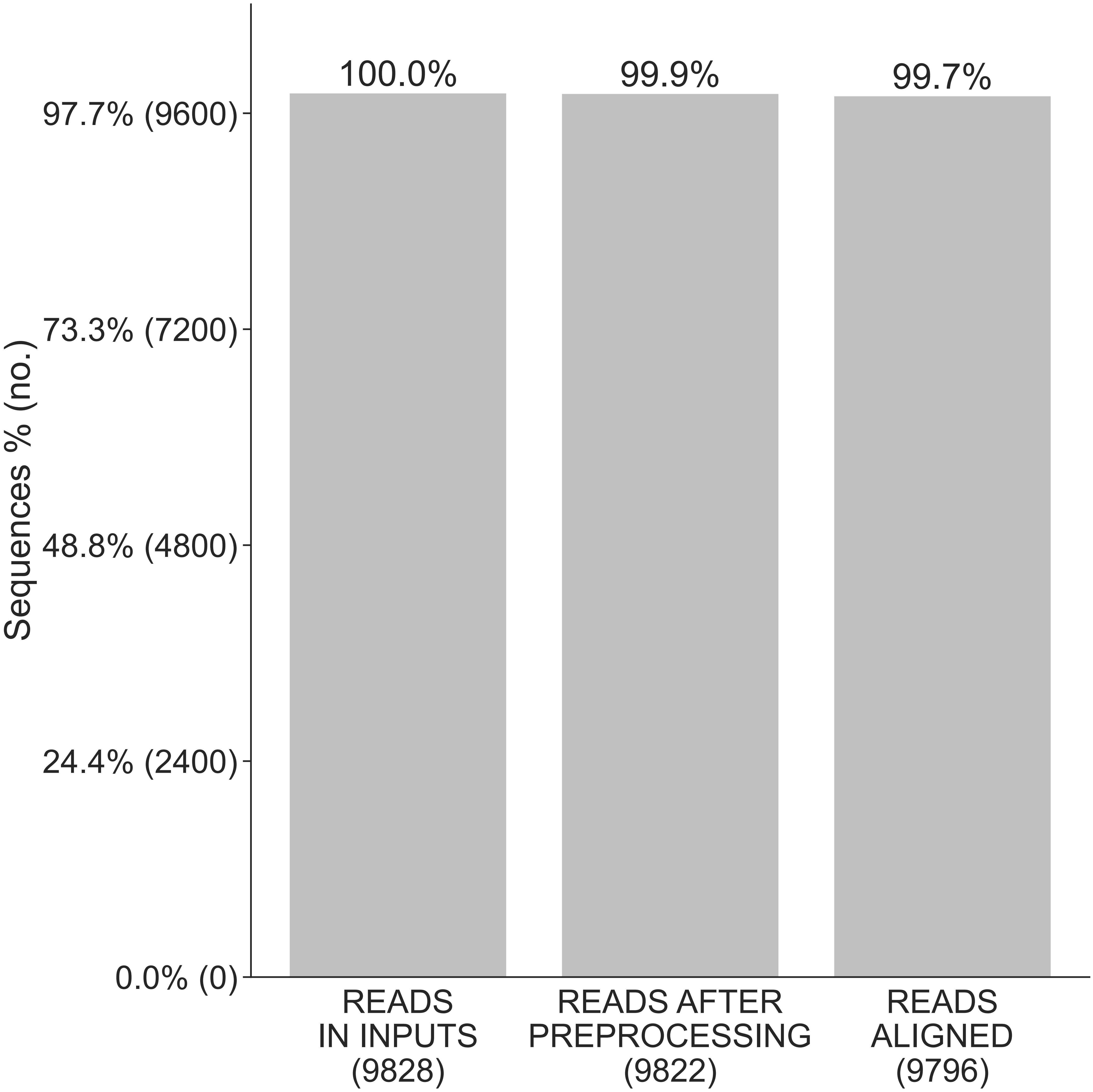
100.0%
99.9%
99.7%
97.7% (9600)
73.3% (7200)
48.8% (4800)
24.4% (2400)
0.0% (0)
Sequences % (no.)
READS IN INPUTS (9828)
READS AFTER PREPROCESSING (9822)
READS ALIGNED (9796)
